# Challenges and enablers to implementation of the Additional Roles Reimbursement Scheme in primary care: a qualitative study

**DOI:** 10.3399/BJGP.2023.0433

**Published:** 2024-04-16

**Authors:** Bethan Jones, Zoe Anchors, Sarah Voss, Nicola Walsh

**Affiliations:** Centre for Health and Clinical Research, University of the West of England Bristol, Bristol.; Centre for Health and Clinical Research, University of the West of England Bristol, Bristol.; Centre for Health and Clinical Research, University of the West of England Bristol, Bristol.; Centre for Health and Clinical Research, University of the West of England Bristol; National Institute for Health and Care Research Applied Research Collaboration West, Bristol.

**Keywords:** additional roles, health plan implementation, primary health care, skill mix, workforce, qualitative research

## Abstract

**Background:**

The Additional Roles Reimbursement Scheme (ARRS) was set up to recruit 26 000 additional staff into general practice by 2024, with the aim of increasing patient access to appointments. Despite the potential benefits of integrating ARRS practitioners into primary care, their implementation has not always been straightforward.

**Aim:**

To explore the challenges and enablers to implementation of the ARRS including its impact on primary and secondary care systems.

**Design and setting:**

Qualitative interview study with ARRS healthcare professionals and key professional stakeholders involved in staff education or scheme implementation across three integrated care systems in England.

**Method:**

Participants (*n* = 37) were interviewed using semi-structured individual or paired interviews. Interviews were audio-recorded and transcribed. Data were analysed using framework analysis until data saturation occurred.

**Results:**

Using framework analysis, 10 categories were identified. Three were categorised as successes: staff valued but their impact unclear; multiple and certain roles maximise impact; and training hub support. Seven were categorised as challenges: scheme inflexibility; creating a sustainable workforce with career progression; managing scope and expectations; navigating supervision and roadmap progression; infrastructure and integration challenges; ARRS roles impact on wider systems; and tensions and perspectives of existing staff.

**Conclusion:**

Most ARRS staff felt valued, but the scheme broadened expertise available in primary care rather than reducing GP burden, which was originally anticipated. Some PCNs, especially those in areas of high deprivation, found it difficult to meet the population’s needs as a result of the scheme’s inflexibility, potentially leading to greater health inequalities in primary care. Recommendations are proposed to optimise the effective implementation of the primary care workforce model. Further research is required to explore administrative role solutions, further understand the impact of health inequalities, and investigate the wellbeing of ARRS staff.

## Introduction

GPs have seen an unprecedented rise in workload pressure in recent years, with greater pressure on primary care services than ever before and insufficient GPs to meet demand.^1^ A more multidisciplinary approach is needed to facilitate enhanced outcomes in patient care and ensure that patients see the most appropriate professional for their needs.^2^ Team-based care in general practice can contribute towards reduced waiting times for appointments and improved staff wellbeing.^3^

One initiative to broaden primary care skill mix in England is the Additional Roles Reimbursement Scheme (ARRS), which was introduced in 2019 and has already delivered its aim of recruiting 26 000 additional staff into general practice by 2024.^4^^–^^6^ NHS England and NHS Improvement reimburse primary care networks (PCNs) for salary and on-costs for a variety of roles ranging from clinical pharmacists to GP assistants. Roles are selected by the PCN to meet local population needs,^7^ and the scheme aims to improve and widen access for patients as well as address workload pressures resulting from primary care workforce shortfall.^4^

Despite the potential benefits of integrating ARRS practitioners into primary care, implementation of the scheme has not always been straightforward or effective.^2^^,^^5^ Challenges previously identified include poor supervision; misunderstanding the scope of roles; a lack of distinct boundaries between roles; and confusion resulting from complex roadmaps (that is, documents that provide educational pathways for those wishing to pursue an ARRS role)^8^ and training guidance.^5^^,^^9^^–^^11^ Many ARRS staff report finding their work rewarding and positive, but they may experience similar stressors to GPs such as large workloads and time pressures.^12^ Some also describe isolation or scope creep.^13^ A key challenge of implementing the ARRS was that PCNs were in their infancy,^5^ so exploration is therefore important. This study aimed to explore the challenges and enablers to implementation of ARRS staff in primary care, including the system’s impact on staff roles and redistribution.

**Table:** How this fits in

The government has delivered on its commitment of recruiting 26 000 more primary care professionals through the Additional Roles Reimbursement Scheme in order to reduce patient waiting lists, widen the range of healthcare services, and meet the needs of local populations. This qualitative study supports the positive impact of these additional roles in broadening the health care available to patients, and finds similar challenges to implementation previously identified (lack of career progression and supervision; lack of understanding of role descriptions and scope creep; problematic roadmaps; and poor integration). However, the data also reveal that the scheme’s inflexibility and lack of available workforce particularly impacted primary care networks in deprived areas, resulting in the potential exacerbation of health inequalities, with the needs of populations not necessarily being met. More flexibility needs to be provided about who and what is funded under the scheme, with particular focus in areas of higher deprivation.

## Method

### Design

This was a qualitative study involving semi-structured individual and paired interviews, with participants recruited against an a priori sampling frame aiming for variation in role, geographical area, and experience to gather a rich overview of the ARRS. All stakeholders were asked broadly similar questions to gather an overview of the scheme, alongside targeted questions about participants’ expertise and experiences in order to address the aims of the study.

### Sampling

Stakeholders whose roles related to implementing the ARRS in three integrated care systems (ICSs) that represented urban, rural, and coastal areas in England were eligible to take part. An a priori sampling frame was used to identify relevant stakeholders including ARRS-funded staff, PCN managers or leads, training hub staff, ICS workforce leads, and staff from organisations involved in recruiting or placing ARRS staff. The sampling frame aimed to capture variation in the sample and enable exploration of the issues from a range of perspectives, as well as allow the researchers to explore specific topics with relevant participants (such as asking more about training with training hub participants). The target sample size was 40–45 participants to collect rich data to answer the research question with sufficient information power.^14^ This is founded on the idea that the sample size for a qualitative study is guided by the study aims, research questions, how specific the sample is, the quality and richness of the data collected, and participants’ relevant experience to the research questions. However, in the event that a smaller number of participants provided enough rich and relevant data based on their experience to meet the research aim, recruitment would cease.

### Recruitment

Snowball sampling was used alongside the a priori sampling frame, with participants encouraged to share the study with colleagues who met the inclusion criteria and matched the sampling frame. Study details were advertised via relevant mailing lists, targeted emails, and the study team’s existing professional networks. Potential participants contacted the research team to be screened against inclusion criteria and participate. Participants provided informed consent verbally or via a form by email.

### Data collection

The study team developed a topic guide based on existing literature and the study aims. This included a list of questions suitable for all stakeholders and specialist questions for specific stakeholder groups (see Supplementary Information S1 for a copy of the topic guide). Two researchers collected the interview data between July and November 2022.

### Analysis

Interviews were audio-recorded and transcribed, and lasted an average of 45 minutes (ranging from 25 to 72 minutes). Transcriptions were de-identified and data were analysed using framework analysis,^15^ which was selected because it can incorporate inductive and deductive analysis to identify patterns within the data, and it is suitable for team-based analyses.^16^^–^^18^ Framework analysis follows a multistep process, and entails familiarisation with data; developing an initial theoretical framework; indexing (organising transcripts into the framework); charting; mapping; and interpretation. Deductive coding was based on the multi-professional framework principles for implementing advanced clinical practice in England.^19^ This framework allowed the researchers to consider which categories were likely to be in the final framework based on topics of interest and to triangulate the creation of the final categories using both the data collected and existing literature. It also assisted in the discussion and refinement of the data categories to ensure they captured answers to the research questions.

## Results

In total, 36 interviews were conducted with 37 participants (one interview was a paired interview) from three ICSs. Table 1 outlines the characteristics of the participants. Framework analysis identified 10 categories from the data. Table 2 presents a summary of the categories, including how each category captured implementation of the scheme at micro, meso, and macro levels, and any staff type differences. Each category discusses successes/enablers of the scheme first, and challenges of the scheme second.

**Table 1.** Qualitative participant characteristics (*n* = 37)

**Characteristic**	***n* (%)**
**Location**	
ICS 1	17 (45.9)
ICS 2	14 (37.8)
ICS 3	6 (16.2)

**Staff role**	
PCN directors	9 (24.3)
Workforce leads^a^	7 (18.9)
Paramedics	4 (10.8)
Pharmacy technicians	4 (10.8)
Social prescribers	3 (8.1)
Care coordinators	2 (5.4)
First contact physiotherapists	2 (5.4)
Clinical pharmacist	1 (2.7)
Health and wellbeing coach	1 (2.7)
Mental health practitioner	1 (2.7)
Practice manager	1 (2.7)
Business manager	1 (2.7)
ARRS project manager	1 (2.7)

**Sex**	
Female	26 (70.3)
Male	11 (29.7)

a *Includes ICS workforce leads, workforce development leads, and training hub roles. ARRS = Additional Roles Reimbursement Scheme. ICS = integrated care system. PCN = primary care network.*

**Table 2.** Success and challenge categories at the micro, meso, and macro levels

**Category**	**Micro (individual level)**	**Meso (PCN level)**	**Macro (ICS/national level)**	**Individual staff type issues**
**Successes/enablers**				
Multiple and certain roles maximise impact	Some staff were working in teams of similar professions rather than alone	Having multiple of a role increased impact	Some roles for example GP assistant easier to evidence impact of the scheme	PC descriptions had overlap and this meant lack of clarity in practice. FCPs had more clarity in role. Paramedics do well with home visits and work that matches previous experience
Training hub support	Training hubs demystify the roadmaps (that arrived late and were unclear) and signposting for training opportunities	Training hubs relieve pressure on PCNs by providing support	Training hubs’ advocation of the scheme in general	Consistent for all staff groups
Staff valued but impact unclear	Most staff are welcomed, valued, and making a difference	Scheme does not necessarily reduce pressure on GPs, but broadens access for patients	Wider concerns about future of scheme, and difficulty in evidencing the benefit because of lack of data, impact of pandemic, and demand increases	Some professions have impact data for example pharmacy technicians. Impact of PC preventive work harder to see
**Challenges**				
Scheme inflexibility	Limitations on eligibility for the scheme, and minimum hourly requirements	ARRS funding does not cover all staffing-related costs so some PCNs use other funding for these	Inequalities within regions because some PCNs can pay above and beyond ARRS caps and some cannot, and therefore lose the funding	Consistent for all staff groups
Creating a sustainable workforce with career progression and retention	Individuals moving roles for career progression or more pay	PCNs planning their workforce based on populations’ needs but often have to recruit pragmatically	Need for pipeline of professionals to work in primary care and ARRS roles, with career progression options	Pre-registration pharmacy placements (to recruit directly)
Managing scope and expectations	Poor understanding of the scope of roles means staff have to manage which patients they can/will see	Clarity about roles can improve implementation. ARRS staff often work differently to GPs	Need for patient education at a broad level to understand the value of roles, and education to help GPs understand the benefit of roles	Need for support for staff but paramedics and PC workers in particular to cope with learning curve and identify scope
Navigating supervision and roadmap progression	Individuals are leaving because of lack of supervision and excessive roadmap requirements	Supervision varies across practices depending on availability of supervisor and attitude towards the scheme	Roadmaps were vague, unclear, and difficult to apply in practice, and suitable courses were lacking	PC workers not only need clinical supervision, but also reflective support and emotional support. Supervision is easier if have space in hierarchy (for example TNAs work well)
Infrastructure, integration, and practical challenges	Working across too many practices in a PCN prevents people feeling part of a team and limits access to estates	Not enough space available for large increase in staff. Variety in success in integrating staff into PCNs	Varied access to infrastructure support such as software (for example, Integrated Clinical Environment) for ARRS staff	Consistent for all staff groups
Impact of ARRS roles on wider systems	Primary care is appealing for work–life balance and work variety	Some work is being held in PCNs because of pressures in other secondary services	Not enough health professionals to meaningfully staff all systems. Rotational models suggested as a solution	Ambulance trusts and mental health service have difficulties in retaining staff — moral implication for recruiting from depleted services
Tensions and perspectives of existing staff	Existing staff may feel undervalued because of losing specialist skills or not getting the same terms and conditions as some ARRS staff	Delicate balance of staff morale between groups of staff with different employment models	Variations in terms and conditions across primary care caused by primary care being private businesses	Registered nurses losing long- term condition care. TNAs and HCAs seeing overlap. GPs losing skills such as joint injections to FCPs

*ARRS = Additional Roles Reimbursement Scheme. FCPs = first contact physiotherapists. HCA = healthcare assistant. ICS = integrated care system. PC = personalised care (for example social prescribing, health and wellbeing). PCN = primary care network. TNA = training nurse associate.*

### Successes and enablers

#### Multiple and certain roles maximise impact

Variation existed in how roles were used and interpreted, and how PCNs maximised their impact. Those who recruited ARRS staff discussed how a single ARRS role was not sufficient, and to maximise the impact, more than one was required:

*‘I think one mental health person on their own is just doomed to fail because you can’t possibly provide any kind of service at all to 50 000 people, it’s hopeless.’*

(PCN lead)

The breadth of the job descriptions also meant that roles differed in different PCNs, depending on local interpretation:

*‘One practice may use a health and wellbeing coach totally different for … weight management or something, exercise. Another one may use them to support pain, addiction to opiates ... ’*

(Workforce lead)

GP assistants and digital transformation leads were additional roles introduced at the end of 2022.^20^ Participants described how these roles could help to maximise the impact of other ARRS roles:

*‘The digital and transformation lead, which is being translated as PCN manager plus. We are going to get one in, because there’s stuff that I just don’t have time for.’*

(PCN manager)

*‘GP assistant is a cross between a super-admin person, and healthcare assistant … and these GP assistants would be a great addition to these teams.’*

(PCN manager)

Participants discussed how expanding the scheme to cover reimbursement for administrative support could increase effectiveness:

*‘Everyone really wants another admin role, and that is someone who’s very highly trained to process test results, results letters, letters to consultants, so that they can start processes off.’*

(PCN manager)

#### Training hub support

The training hubs^21^ were considered crucial for demystifying training pathways or providing guidance to staff in all roles:

*‘*[Professional lead] *has been an absolute champion for just getting in touch with those silly little questions, queries, and then knowledge and information that* [they] *can provide about the whole process.’*

(Paramedic)

*‘I don’t think you can undervalue the role that the training hub could play, and do play.’*

(Training hub lead)

#### Staff valued but impact unclear

Most ARRS staff participants felt welcomed and valued, and believed that ARRS roles were making a difference. While participants understood that the scheme’s original aim was to reduce the burden on GPs, many acknowledged that pressures on primary care meant that this had not yet been realised. Instead, participants described the broadening of services and an increase in the quality of care:

*‘There’s an improvement in the quality of the care that the patients are getting because someone with far more knowledge is on that specific case.’*

(Workforce lead)

However, there were circumstances where participants did describe ARRS staff saving GPs time and resources:

*‘I think the pharmacists have really taken workload off of the GPs, and also the pharmacy technicians in terms of what they’ve taken off some of the admin side of things.’*

(Practice manager)

While interviewees perceived a positive impact, it was hard to demonstrate, particularly given the external factors impacting primary care. The impact of the scheme was also influenced by its uncertain future; some participants described how these concerns impacted engagement with the scheme:

*‘Initially, my PCN was very reluctant because they were scared of what would happen if the funding wasn’t there. Would the five surgeries have to cover our costs?’*

(Workforce lead)

Staff working in ARRS roles also expressed concerns about their job security:

*‘I don’t know how long the ARRS funding will last because I know it’s like 23–24. There’s this break point and you think, “Oh, does that mean I’m not going to be employed after that?”’*

(Clinical pharmacist)

Despite difficulty in evidencing impact and a mixed picture relating to the benefit to GPs, participants were strongly in favour of continuing with the scheme:

*‘There would be uproar, absolute uproar if* [ARRS roles] *were taken away.’*

(Workforce lead)

### Challenges of the scheme

#### Scheme inflexibility

Most participants described the inflexibility of the scheme. They discussed how the ‘one size fits all’ nature limited PCNs’ abilities to fully engage with the scheme:

*‘It’s a very rigid scheme and as we’ve gone through the years there’s been more and more pressure for flexibility*.*’*

(Training hub lead)

Participants described how the scheme did not cover many costs associated with recruiting and retaining staff, for example, costs for supervision, costly and inconsistent third party recruitment tariffs, estates, and pay uplifts. The rigid salary scales also led to PCNs having to find extra funding for additional costs or to make roles attractive. Not all PCNs could afford this:

*‘*[Healthcare organisation] *just put out 10 jobs for Band 5 and Band 6* [pharmacy] *technicians at £33,000 and £40,000. We can’t get anywhere near it ... ’*

(Pharmacy technician)

The inflexibility of the scheme’s funding was thought to exacerbate inequalities because PCNs that could not afford the additional costs associated with hiring an ARRS role, or that were unable to recruit, ended up not using the full funding. Unused funding was returned to a central pot, and made available to other PCNs who could use it:

*‘So, it is that lack of flexibility really that stops us using all our money, which seems a shame because it’s just going back in some central pot somewhere.’*

(PCN lead)

*‘It’s damaging, and increasing health inequalities, because we have identified a real need for a physio, it isn’t the 15 roles available to us* [financially]*, we are locked out of being able to provide one ... ’*

(PCN manager)

#### Creating a sustainable workforce with career progression and retention

Participants demonstrated the need for a sustainable pipeline of professionals to work in ARRS roles, including career progression opportunities for existing staff, and employment models with other organisations to retain staff. However, the ARRS does not build in sufficient career progression:

*‘One of the other challenges is that there is no kind of entry level pharmacist role in general practice.’*

(Workforce lead)

Some participants discussed how ARRS staff left roles because of a lack of career progression, particularly in certain roles:

*‘You don’t keep pharmacists for 2 minutes. As soon as they finish the pathway they’re gone.’*

(Pharmacy technician)

Participants also used rotational models to sustain the primary care workforce in the long term:

*‘We now do a rotation scheme. So, they spend so many months in the hospital, so many months in a community pharmacy, and so many months in a GP practice.’*

(Workforce lead)

When discussing how PCNs specifically planned and recruited their ARRS workforce, PCNs made strategic decisions to meet their population needs and recruit staff. However, sometimes workforce design was more *‘piecemeal’*:

*‘We do have a vision that one day we will have a team … I think instead of it growing up piecemeal the way it has, it would have been lovely to just sit down with a blank piece of paper and draw up a plan.’*

(PCN manager)

PCN staff in deprived or rural areas sometimes had to make more pragmatic recruitment decisions to use funding, recruiting based on availability rather than need:

*‘The plan is to have more pharmacists but where I’m not able to recruit the pharmacists … do I then sit there and do nothing or do I use that funding and take it somewhere that actually I can recruit and make the most out of that area?’*

(PCN manager)

#### Managing scope and expectations

Some ARRS staff reported that colleagues’ understanding of their roles varied, despite this understanding being key to the scheme’s success. Defining scope was particularly challenging for the personalised care roles:

*‘How is a health and wellbeing coach different to a social prescriber? How is a social prescriber or a health wellbeing coach different to a care coordinator?’*

(Workforce development lead)

This understanding is crucial because it impacted how ARRS staff were used:

*‘You get your GPs, and your clinicians that get it and refer in all the time … To other ones that you never get a referral from, however, you know full well they will be seeing patients that we could be helping.’*

(Social prescriber)

Poor understanding also derived from a lack of appreciation that roles may not suit traditional GP appointment structures. This was particularly relevant for social prescribers, who often required longer appointments when dealing with complex psychosocial issues:

*‘One of the difficulties I face as a link worker is having to justify my existence to a lot of people … they’re like “Why can’t you see 50 people a day?”, “Why don’t you do what a GP does?”.’*

(Personalised care workforce lead)

Participants described their role to aid colleagues’ understanding:

*‘We had a new pharmacy manager … we actually put down the stuff that we can do, she was surprised and she forwarded it to the lead clinician and he had no idea that’s what we could do.’*

(Pharmacy technician)

Similarly, ARRS staff themselves struggled if they were not adequately prepared for the challenges of primary care:

*‘Very often paramedics are really undereducated on what primary care really is, and how hard it is, and how complex it is, and how intense it is.’*

(Paramedic training hub lead)

The steep learning curve experienced by those working in primary care raised the importance of having a clear scope and boundaries for each role. Participants discussed their concerns around scope creep:

*‘I think one thing you do have to be careful with the roles as well is to define the boundaries of the roles … With practices being under significant pressure, I think there’s obviously the risk of scope creep on some of these roles.’*

(Workforce development lead)

#### Navigating supervision and roadmap progression

For the ARRS to succeed, staff need adequate supervision and training, which is guided by roadmaps under the scheme. However, much of this guidance arrived after the roles had been established, and roadmap documents were unclear:

*‘If you read the roadmap it makes no sense, it’s really confusing, it’s like, where do you start?’*

(Paramedic workforce lead)

Some viewed the roadmaps as a useful, structured approach for novices entering primary care. However, for others, there was a risk of duplicating past training or experience unnecessarily:

*‘There is an exemption process, which is very arduous, and I have known a couple of pharmacists that have gone through that, but it doesn’t seem to account for those that have got a lot of experience elsewhere.’*

(Pharmacy workforce lead)

Supervision of ARRS staff varied and some staff felt their day-to-day supervision was too sporadic, and wanted more regular support:

*‘I also had one of the GPs who’s my mentor. We were having monthly one-to-one sessions but that has gone by the by a bit … I’m very keen to get those back up.’*

(Care coordinator)

#### Infrastructure, integration, and practical challenges

A consistent challenge for PCNs was the lack of space to accommodate ARRS staff:

*‘Space is a real challenge, we have kind of had this influx of all of these opportunities for these other roles, but nowhere to put anybody.’*

(Workforce development lead)

Another consideration was working across multiple sites, and staff working in this way found it difficult to integrate into teams:

*‘Because you cover so many surgeries, you are dipping in and dipping out. So, you do get to know people, but really on a very, very surface level.’*

(Mental health practitioner)

Those who were employed by the PCN risked getting lost in *‘limbo’* because of their employment status, as one participant explained:

*‘ARRS staff are just stuck in limbo, so they don’t belong to a practice … it’s very difficult to make them feel that they belong to the PCN.’*

(Business manager)

Integration challenges were not the case for all, however:

*‘Being integrated with the GPs has been really helpful for me because I think I’m learning so much working amongst them, and you don’t feel like an outsider coming in.’*

(Pharmacy technician)

Some ARRS staff discussed the benefit of working in hubs, where professionals in the same role across a single PCN were working in one location:

*‘Because we’re over the six practices — it would be really, really good to have a hub, a base for us three. So, because we are a PCN, we should all be working together.’*

(Advanced lead social prescriber)

#### Impact of ARRS roles on wider systems

There were unintended consequences at system-wide levels, including large numbers of staff moving from other services to work in the scheme, which left some services depleted of their workforce:

*‘I suppose there might be in terms of destabilising because if you’ve got a lot of PCNs trying to recruit paramedics, for example, and there aren’t enough paramedics to go round … I guess that’s a risk that we’re all fishing in the same pond for staff.’*

(PCN manager)

Some staff chose to move into primary care for personal development or because they saw a better work–life balance and more social working hours:

*‘*[In hospital] *you used to have to cover a late shift and they don’t do that here … there’s a lot of pluses for a tech leaving secondary care to come here.’*

(Pharmacy technician)

A potential positive impact of the scheme was that patients with issues previously requiring referrals could be managed in primary care:

*‘So,* [staff in primary care] *stops those referrals going in, which is good. And, it’s the same for the physio service. That certainly stops the referrals into the main physio service.’*

(ICS lead)

However, pressures elsewhere in the system (potentially exacerbated by the movement of staff into primary care) meant that primary care staff were supporting issues that they might not otherwise. This was particularly challenging for social prescribers:

*‘We are hand-holding due to the lack of resources everywhere else and availability on other agencies. We’re hand-holding some very, very serious stuff.’*

(Advanced lead social prescriber)

#### Tensions and perspectives of existing staff

A delicate balance is needed to incorporate additional staff into primary care without demoralising or deprioritising existing staff. There were concerns that some staff in primary care, particularly registered nurses, missed out on using their specialist knowledge because that work was being covered by incoming ARRS staff:

*‘*[Nursing staff] *saw some of these roles coming in and taking away their work. They, therefore, felt demoralised, they felt that there was no value placed on the work that they had been doing for years … and it’s all been given to a clinical pharmacist to sort out.’*

(ICS lead)

The difference in terms and conditions between ARRS and non-ARRS staff were also causing tensions, as well as disparities in opportunities in primary care more broadly:

*‘This is one of the problems as our team see it; the additional roles are paid much more than anyone else in primary care.’*

(PCN manager)

## Discussion

### Summary

This study investigated the implementation of the ARRS scheme, which aimed to meet the rising demand on primary care, expedite patient access, and provide a career pathway for ARRS practitioners, in terms of its successes and challenges. Success was evident in terms of ARRS staff feeling valued and providing a range of care to patients; however, the reduction of workload pressures resulting from a shortfall in the primary care workforce has yet to be realised. Several enablers to the scheme were noted, along with implementation challenges that have previously been identified and still continue to be a problem. The scheme’s impact was maximised when PCNs deployed multiple roles of one ARRS type. The study identified a new challenge of scheme inflexibility, which highlighted that PCNs (particularly in areas of high deprivation) were often prevented from meeting the needs of their population. It is important to provide more funding flexibility to prevent further widening of health inequalities in primary care. Other challenges identified were lack of career progression and supervision; limited understanding of role descriptions and scope creep; problematic roadmaps; and poor integration and unintended consequences (on secondary systems and existing primary care staff).

### Strengths and limitations

A strength of this study is exploration of the impact of the ARRS on the system (primary and secondary care). Workforce leads, PCN directors, and practice managers as participants were asked about the impact of the ARRS on the healthcare system and to consider the ARRS roles as a whole rather than the different groups in isolation. The study found that the incoming skill mix of staff was not directly (as yet) impacting workload pressures in primary care, but instead broadening the types of health care available to primary care patients. This is similar to what has previously been revealed for advanced clinical practitioner roles.^22^

This study has three key limitations. First, findings are limited to only three ICSs and provide a snapshot in time that could quickly become outdated given NHS policy changes in a comparatively new and evolving scheme. Second, the interpretation relies on qualitative methodology and further quantitative investigation would be valuable to evaluate the impact of ARRS roles. Linked PCN and GP workforce datasets are currently being analysed to understand the effectiveness of the scheme.^23^ Third, GP and patient participants were not included in this study. Their views have been explained elsewhere, with GP and patient satisfaction not necessarily increasing with skill mix changes.^24^

### Comparison with existing literature

Identified challenges are similar to those identified in existing research (for example, lack of supervision, career progression opportunities, and infrastructure),^5^^,^^10^^,^^25^^,^^26^ which indicates that these issues are persisting. Less attention has been given to the scheme’s inflexibility and its impact on widening health inequalities. The current study did not find that all PCNs were able to select and recruit staff based on their population needs, which was an original aim of the scheme.^7^ Indeed, some argue that although ARRS roles could inform how primary care staff can collaborate to reach underserved populations,^27^ PCNs have lacked a clear strategy and ‘buy-in’ for the ARRS roles.^5^ This study found that PCNs had strategies, but the scheme’s inflexibility often prevented them from implementing them, particularly in areas of high deprivation.

### Implications for research and practice

This study has several implications for policymakers. Consideration should be given to building a sustainable workforce with career progression opportunities for staff, including rotational working to avoid impeding secondary systems. ARRS staff would also benefit from clearer roadmaps and recognition of accredited learning, allowing experienced staff to complete more efficient pathways. Professional leads at training hubs have been invaluable in assisting with navigating roadmaps and should continue. Supervision is currently suboptimal for many ARRS staff. Although this may improve as more staff join the scheme, increasing supervision capacity and options to follow university training pathways would address the current lack of practice-based supervision. If viable, setting up hubs for similar ARRS roles would assist in addressing infrastructure and integration issues, as would limiting the number of practices in which a staff member works.

If possible, greater flexibility is needed regarding who and what is funded under the scheme. A sum of money to cover incidentals for hiring staff or to provide pay uplifts would assist many PCNs that may be struggling to recruit ARRS staff. Furthermore, as third party recruitment tariffs were revealed to be problematic and not covered by the scheme, fee negotiations with these organisations should be held at a broader level with the aim of making these consistent and fair.

Further research would be useful to explore the viability of certain policy recommendations detailed above, such as consultation with key stakeholders in secondary systems (such as ambulance or mental health trusts) to develop future solutions to staff depletion. Research should focus on the existence of workforce provision inequalities, with particular focus on the recruitment and retention of ARRS roles in areas of high deprivation.

Several challenges have been identified that potentially compromise the wellbeing of ARRS staff. Social prescribers faced many challenges, particularly in urban areas, and felt their impact in primary care was not valued. Further research into this staff group and monitoring of the wellbeing of ARRS staff in general would therefore be worthwhile. Finally, this study revealed that administrative roles were highly desirable in the ARRS, with much interest in the new GP assistant role. Investigation of this role as a viable solution to heavy GP workloads would be valuable as the impact of administration is often neglected in policy making.
